# The Double Face of Ketamine—The Possibility of Its Identification in Blood and Beverages

**DOI:** 10.3390/molecules26040813

**Published:** 2021-02-04

**Authors:** Magdalena Świądro, Paweł Stelmaszczyk, Irena Lenart, Renata Wietecha-Posłuszny

**Affiliations:** Laboratory for Forensic Chemistry, Department of Analytical Chemistry, Faculty of Chemistry, Jagiellonian University, 30-387 Kraków, Poland; magda.swiadro@doctoral.uj.edu.pl (M.Ś.); pawel.stelmaszczyk@doctoral.uj.edu.pl (P.S.); irena.96.lenart@gmail.com (I.L.)

**Keywords:** dried blood spot, ketamine, CE-TOF-MS, date-rape pills

## Abstract

The purpose of this study was to develop and validate a high-sensitivity methodology for identifying one of the most used drugs—ketamine. Ketamine is used medicinally to treat depression, alcoholism, and heroin addiction. Moreover, ketamine is the main ingredient used in so-called “date-rape” pills (DRP). This study presents a novel methodology for the simultaneous determination of ketamine based on the Dried Blood Spot (DBS) method, in combination with capillary electrophoresis coupled with a mass spectrometer (CE-TOF-MS). Then, 6-mm circles were punched out from DBS collected on Whatman DMPK-C paper and extracted using microwave-assisted extraction (MAE). The assay was linear in the range of 25–300 ng/mL. Values of limits of detection (LOD = 6.0 ng/mL) and quantification (LOQ = 19.8 ng/mL) were determined based on the signal to noise ratio. Intra-day precision at each determined concentration level was in the range of 6.1–11.1%, and inter-day between 7.9–13.1%. The obtained precision was under 15.0% (for medium and high concentrations) and lower than 20.0% (for low concentrations), which are in accordance with acceptance criteria. Therefore, the DBS/MAE/CE-TOF-MS method was successfully checked for analysis of ketamine in matrices other than blood, i.e., rose wine and orange juice. Moreover, it is possible to identify ketamine in the presence of flunitrazepam, which is the other most popular ingredient used in DRP. Based on this information, the selectivity of the proposed methodology for identifying ketamine in the presence of other components of rape pills was checked.

## 1. Introduction

Ketamine was synthesized for the first time in 1961–1963 by Calvin Stevens in the Parke-Davis laboratory in Michigan. A few years later, ketamine was patented as an anesthetic for both humans and animals [1]. As well as being an anesthetic, it also serves as an analgesic, for example in the treatment of persistent post-surgical pain [2,3]. Moreover, the S-enantiomer of racemic ketamine is approved for treatment-resistant depression and for use in depressed patients with acute suicidal ideation or behavior [4]. However, some of the effects and properties of ketamine have led to its abuse for criminal purposes: it causes drowsiness, hallucinations, amnesia, impaired coordination, and general fatigue, as well as confusion and memory loss [5], and furthermore, it is a colorless, tasteless and odorless liquid, and thus difficult to detect. It is one of the main ingredients, alongside flunitrazepam and gamma-Hydroxybutyric acid (GHB), in so-called “date-rape” pills (DRP).

In response to this threat, several innovative methods have been developed to detect these substances quickly and easily in drinks or meals. Nowadays, the most popular test is a low-cost, “naked eye” assay indicating the presence of DRP in alcoholic and other beverages, based on the colour change of paper sensors, proposed by Narang et al. [6].

Yehia et al. [7] have developed a microfluidic device with which to combine potentiometric, fluorometric, and colorimetric detection zones. This newly developed trimodal paper chip has been used for on-site determination of ketamine hydrochloride (KET) as a date rape drug in beverages [7].

The proposed tests or devices for identifying the main ingredients such as ketamine in DRP do not always produce a reliable result—discoloration can be influenced by other substances in the matrix. Therefore, the proposed tests should be constantly verified with reference methods. Ketamine and the other ingredients in the DRP can be determined in human blood and urine, as well as in drinks using the liquid chromatography method coupled with a mass spectrometer (LS-MS) or molecularly imprinted solid-phase extraction (MISPE) [8]. These techniques should be fast, have high precision, and be microinvasive, thereby increasing the comfort of sampling and, furthermore, be consistent with the principles of green chemistry. Therefore, the authors of the present research proposed use of the Dried Blood Spot (DBS). It is an innovative method that offers a simple and practical means of blood collection—making it an attractive alternative to traditional methods. The DBS technique has several advantages over conventional ones for various biological materials such as blood, plasma, or serum. This method is microinvasive—collected through finger or heel pricks. The volume of blood required is smaller—less than 50 µL. Blood samples spotted on a DBS card can be stored for several months or years at room temperature. Due to these advantages, DBS is mainly used in forensic toxicology, drug screening, therapeutic drug monitoring and neonatal screening [9,10,11].

The DBS method is usually combined with liquid chromatography, but it could be further investigated in combination with capillary electrophoresis, which was proposed by Świądro et al. [10]. Capillary electrophoresis (CE) is characterized by simple instrumentation, high separation efficiencies, short analysis times, and minimum consumption of reagents and samples, which is in accordance with the principles of green chemistry. Moreover, CE has great potential for miniaturization and portability. These characteristics are of particular interest in biomedical and forensic applications, where a rapid analytical response is required, and the amount of samples is often limited, and, furthermore, analysis should preferably be performed at the scene of forensic investigations [10,12].

The purpose of this research was to preliminary test for identification ketamine in human blood samples and qualitatively in various types of beverages, i.e., orange juice, rose wine, and Coca-Cola. To determine ketamine, DBS cards were used, on which drops of human blood and beverages were applied. Discs were cut from the DBS cards, which were subjected to microwave-assisted extraction (MAE), which, combined with capillary electrophoresis and coupled with a mass spectrometer (CE-TOF-MS), allowed final results to be obtained successfully. This may be the best preliminary research on the optimization of professional techniques for identifying ketamine in different matrices. In addition, it is important background research on the preparation of an innovative device based on portable micro-capillary electrophoresis.

## 2. Results and Discussion

### 2.1. Validation Parameters

**Figure 1:**
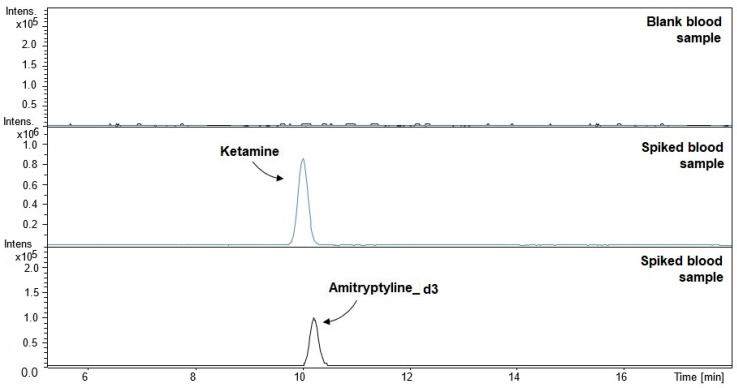
Electropherograms of blank and spiked blood samples (ketamine at a concentration of 150 ng/mL: t_m_ = 10.0 ± 0.6; amitriptyline-d3 at a concentration of 150 ng/mL: t_m_ = 10.1 ± 0.5).

The DBS/CE-TOF-MS method, in combination with MAE extraction with ethyl acetate, at the sample preparation stage and the use of a background electrolyte composed of 100 mM formic acid: acetonitrile (90:10, *v/v*) at the separation stage enabled detection of ketamine in blood samples. Figure 1 shows the obtained electropherograms of blank and spiked blood samples.

Blood of unknown hematocrit was used in the present research. Some strategies to eliminate the influence of the hematocrit level on the results of quantitative analysis presented in publications suggest the use of special pipettes, early cutting of the discs from DBS cards and the use of a special DBS cartridge [13,14]. Applying the strategy proposed by Majda et al. [15] allows the use of one blood type (with an unknown hematocrit value) and the calculation of the volume of blood cut from DBS cards. This method allows you, using simple tools and calculations, to relate the result obtained for real blood samples of unknown hematocrit to the determined calibration curve. Therefore, Majda’s calibration strategy was used. Each blood drop was the same: average diameter: 7.9 ± 0.1 mm, DBS area: 97.5 ± 0.1 mm^2^, cut out surface area: 56.5 mm^2^, average cut out volume: 30.0 ± 0.2 µL. Therefore, only the final results have been presented in Table 1 (validation parameters).

The developed method is characterized by a linear dependence of concentration on the measured signal in the range of 25–300 ng/mL. The R-squared of the established calibration curve is 0.997, which is satisfactory with regard to acceptance criteria in European Medicines Agency (EMEA) [16] and Scientific Working Group for Forensic Toxicology (SWGTOX) guidelines [17].

Values of limits of detection (LOD) and quantification (LOQ), calculated from the calibration curve, were 6.5 ng/mL and 19.5 ng/mL, respectively. Values of LOD and LOQ were determined based on the signal-to-noise ratio were 6.0 ng/mL and 19.8 ng/mL, respectively. Both parameters determined by these two methods are approximately equal, which shows that the method depending on calibration curve parameters is useful for establishing values of LOD and LOQ, before determining these parameters in an experimental way. The method based on calibration curve parameters was used to estimate the order of magnitude, which was helpful in planning the experiment to determine the LOD and LOQ values based on signal-to-noise ratio. Bearing in mind the small amount of blood sample applied on DBS cards, the obtained values could be considered highly satisfactory. Odoardi et al. [18] using the DBS method coupled with UHPLC-MS/MS reported an LOD for ketamine of 0.5 ng/mL, using 90 µL of sample and cutting out a 3 mm diameter disk from three spots. Another work based on DBS-LC-MS/MS reported an LOD equal to 1.0 ng/mL, using 10 µL of sample and cutting out an entire blood spot [19]. The method proposed in this manuscript is less sensitive than DBS-LC-MS. However, a significant advantage of our method is the small amount of blood sample needed for analysis. Modification of stages of the analytical procedure and their parameters (e.g., the area cut from DBS cards, volume of extraction solution or MAE extraction parameters) would probably increase the sensitivity of the method in future research. Our results show that CE-MS can be used as an efficient substitute for LC-MS methods. In research by Nosseir et al. [20], 10 ng/mL was determined as the LOQ using the LC-MS/MS method and 100 µL of sample. In other studies, such as one by Rosas et al. [21], the LOQ was 1 ng/mL using the LC-MS method, or in research by Hasan et al. [22], the LOQ was 0.1 ng/mL using the LC-MS/MS method. However, their procedures used much larger blood volumes of 450 µL and 200 µL, respectively. These are 9-times and 4-times higher than in our procedure, respectively. Comparing it to our results, in which the LOQ is higher, the advantage of our method is a smaller sample volume. If a similar amount of sample was used for analysis in our method, it would be possible to obtain a similar limit of quantification. Additionally, in research by Legrand et al. [23] with the use of a C18 micro column and the LC-MS method, it was possible to achieve an LOQ of 4 ng/mL using a blood sample in the amount of 100 µL—comparing this to our results the LOQ obtained by us is higher, but by increasing volumes of blood in our procedure we would achieve a lower LOQ than by Legrand et al. [23]. This indicates that lower limits of quantification can be achieved by using DBS/MAE/CE-TOF-MS than by using some developed procedures involving the LC-MS method.

Precisions on each determined level were in the range of 6.1–11.1% for one day and in the range of 7.9–13.1% over 3 consecutive days. The recoveries of the developed method had the widest range for low concentrations, which were 92.7–111.3% of the nominal concentration. According to SWGTOX acceptance criteria, the coefficient of variation-CV% should be ±15.0% for medium and high concentrations and ±20.0% for low concentration; recovery should be in the range of 80.0–120.0%. The developed method was characterized by satisfactory precision and recovery on each considered level, and the results of the research demonstrate its capability to determine ketamine in blood samples with good recovery and precision.

### 2.2. Analysis of Beverage Samples

**Figure 2:**
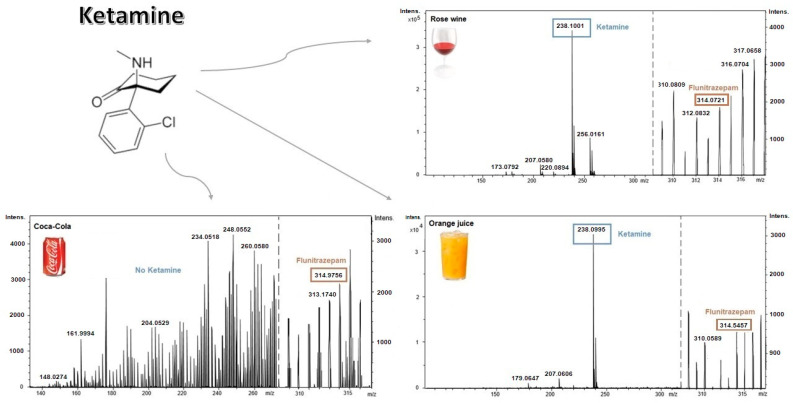
The mass spectra for the beverages—rose wine, orange juice, and Coca-Cola—containing ketamine.

The developed analytical procedure was tested by applying it in the qualitative analysis of three types of beverages: orange juice, Coca-Cola, and rose wine. Due to the fact that flunitrazepam as well as ketamine is used as an ingredient in DRP, the possibility of detecting ketamine in the presence of flunitrazepam was tested. Beverages containing ketamine were put on DBS cards. Dried samples were analyzed according to the developed procedure for blood. The mass spectra recorded by the detector for the tested samples are shown in Figure 2.

The results indicate the effectiveness of the developed method as a preliminary test for the identification of investigated drugs in some beverages. The method is selective for flunitrazepam in both blood and beverage samples. Coca-Cola is the only drink in which no ketamine was detected in the presence of flunitrazepam. However, current research by, e.g., Albright et al. [24] and Mohamed et al. [25] suggests no interference in the detection of ketamine from the matrix in the Coca-Cola drink. Furthermore, since both drugs together have been detected in juice and wine, the presence of flunitrazepam does not prevent ketamine from being detected. However, one possible scenario is that constituents of the Coca-Cola mask its presence in the beverage under extraction conditions. This aspect should be carefully studied in future research.

## 3. Materials and Methods

### 3.1. Chemicals and Reagents

Flunitrazepam, ketamine, and amitriptyline-d3 (deuterated), which was used as an internal standard, were purchased from Lipomed AG (Arlesheim, Switzerland) at a concentration of 1 mg/mL. All samples were stored as frozen at −20 °C before analysis, according to standard laboratory practice. The other reagents used throughout the experiments were: 30% NaOH water solution (Avantor Performance Materials, Gliwice, Poland), technical nitrogen with 90–99% purity (Air Products, Poland), and ultrapure water (18.2 MΩ cm, less than 3 ppb TOC) generated with the Milli-Q system by Merck-Millipore (Darmstadt, Germany). Formic acid, methanol, and acetonitrile were obtained from Sigma–Aldrich (>99%, St. Louis, MI, USA). In addition, in the research: Coca-Cola by The Coca-Cola Company^©^ (USA), orange juice by Tymbark-MWS Sp. z o.o. S.K.A. (Poland), and rose wine by Carlo Rossi (USA), were used as matrices for the determination of the analyzed substances.

Whatman FTA DMPK C cards (not chemically impregnated) and a Harris Uni-Core 6-mm puncher were purchased from Sigma–Aldrich (St. Louis, MA, USA). Electrophoresis vials (1.5 mL), and inserts (200 μL) were purchased from VWR (Randor, USA). Eppendorf vials (1500 and 5000 μL) were produced by Eppendorf AG (Hamburg, Germany).

### 3.2. Instrumentations

The CE–MS experiments were carried out using a PA 800 plus capillary electrophoresis system (Beckman Coulter, Brea, USA) coupled to a MicrOTOF II mass spectrometer (Bruker, Bremen, Germany) with electrospray ionization source (ESI) and time of flight analyzer (TOF). A background electrolyte (BGE) of 100 mM formic acid:acetonitrile (90:10, *v/v*) was used. Fused- silica capillaries (Beckman Coulter, Brea, USA) had a total length of 100 cm and an internal diameter of 75 μm. The separation process was carried out for 25 min. Hydrodynamic injections were performed using a pressure of 4.83 kPa (0.7 psi) for 6 s. The separation voltage was 30 kV and the capillary temperature was set to 25 °C (capillary) and to 15 °C (sample storage). The sheath liquid was delivered with a flow rate of 180 μL/h using a syringe pump (KD Scientific, Holliston, USA). The MS parameters were as follows: nebulizer pressure: 0.4 bar; capillary voltage: 4500 V; dry gas: 0.4 L/min heated to 180 °C. The positive ionization mode was used and data were acquired in the mass range of 100–1450 *m/z*. Sodium formate clusters were used for internal calibration, according to the procedure described by Bruker. Data were acquired and processed by Compass Data Analysis 3.2 software (Bruker, Bremen, Germany).

A MARS 5 microwave-assisted sample preparation system (CEM, Matthews NC, USA), equipped with 24 Xpress^®^ PFA vessels (20 mL), was used for the extraction of flunitrazepam and ketamine from human blood, Coca-Cola, orange juice, and rose wine. The diameters were measured using an electronic caliper (Magnusson).

### 3.3. Procedure

The ketamine spiked blood samples were prepared at concentrations of 50, 100, 150, 200, 250, and 300 ng/mL. Each standard blood sample contained amitriptyline-d3 as an internal standard (IS) at a concentration of 150 ng/mL. Appropriate amounts of ketamine and IS standard solutions were pipetted into successive Eppendorf vials and dried under nitrogen gas. Next, 50 µL of blood with unknown hematocrit, which did not contain the ketamine, was added to each vial with dried residue and vortexed. The spiked blood samples were applied on the FTA DMPK C DBS cards as two drops (25 µL each) and subsequently dried for 1.5 h at room temperature. Next, two DBS discs created from two drops were cut out using a puncher (6-mm diameter disc) and put into vessels (25 mL) containing 1.6 mL of 0.6 M NaOH and sonicated for 5 min. Then, 3 mL of ethyl acetate was added and extraction was carried out using the microwave-assisted extraction (MAE) procedure. MAE extraction was as follows: 16 min at a temperature of 55 °C using microwave power ranging from 480 to 800 W. In the next step, 2.5 mL from each vessel was transferred to a plastic conical tube and dried under nitrogen gas at a temperature of 40 °C. Next, 500 μL ethyl acetate was added and centrifuged for 5 min (4000 rpm, 4 °C), and then the mixture was again dried under nitrogen at 40 °C. Finally, the residue was dissolved in 50 µL of 100 mM formic acid:acetonitrile (90:10, *v/v*)—100 times diluted. The prepared samples were analyzed using capillary electrophoresis coupled with a mass spectrometer (CE-TOF-MS).

### 3.4. Validation Parameters

The method was validated on whole blood samples based on recommendations issued by the European Medicines Agency (EMEA) in the Guideline on bioanalytical method validation [16], and by the Scientific Working Group for Forensic Toxicology (SWGTOX) in Standard Practices for Method Validation in Forensic Toxicology [17]. The guidelines do not contain specific regulations for the validation of the DBS method. The procedure of sample preparation (including blood drop volume, drying time) was developed based on the suggestions of International Association for Therapeutic Drug Monitoring and Clinical Toxicology in the Guideline on development and validation of DBS methods [26]. In order to avoid the influence of (unknown levels of) hematocrit, the same type of blood and calculation strategy were used as in the work of Majda et al. [15]. Validated parameters were linear range, limit of detection (LOD), limit of quantification (LOQ), specificity, intra-day precision and recovery, inter-day precision and recovery. All measurements were carried out using blood samples that were free of ketamine and internal standard. In future research, it will be necessary to perform a full method validation considering, apart from the basic validation parameters, also parameters specific to DBS methods such as volume effect and Volcano effect [26].

#### 3.4.1. Linearity Range

Linearity was studied in the range of 25–300 ng/mL using spiked blood at 7 levels (25, 50, 100, 150, 200, 250, and 300 ng/mL). The calibration curve was drawn up as the correlation of the ketamine concentration in spiked blood with the signal, which was equal to the ratio of the peak surface areas (KET/IS). Each sample contained amitriptyline-d3 added as an internal standard in a concentration equal to 150 ng/mL. The chemical structures of the two compounds are different but they have an identical retention time. The coefficient of determination (R-squared) was calculated by the least squares method.

#### 3.4.2. Limits of Detection (LOD) and Quantification (LOQ)

The limit of detection (LOD) and limit of quantification (LOQ) were estimated by the ratio of the standard deviation of the intercept factor to the slope of the calibration curve, according to Equation (1).

(1) $$\mathrm{LOD}\;\left(\mathrm{LOQ}\right)=\;\frac{x\cdot \mathrm{SD}}{b}$$

**Figure 3:**
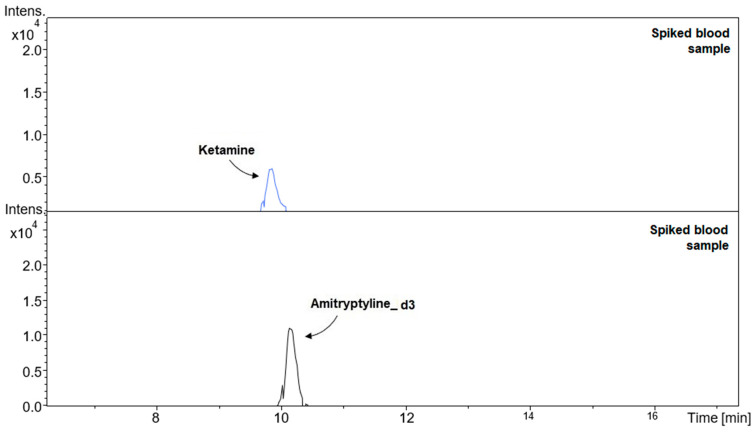
Electropherograms of spiked blood samples (ketamine at the LOQ level—20 ng/mL: t_m_ = 9.08 ± 0.4; amitriptyline-d3 at a concentration of 150 ng/mL: t_m_ = 10.3 ± 0.5).

In Equation (1): x is constant (for LOD it is equal to 3.3, for LOQ it is equal to 10); SD is the standard deviation of the intercept factor of the calibration curve; b is the slope of the calibration curve. The estimated LOD value was confirmed experimentally using the signal-to-noise ratio. In this method, LOD was determined by analyzing at least five samples at different concentrations of ketamine: 2, 6, 10, 15, and 20 ng/mL. The lowest concentration at which the signal-to-noise ratio was equal to 3 was taken as the LOD. The LOQ was considered to be 3.3 times higher than the determined LOD. Figure 3 shows electropherograms of spiked blood samples at the LOQ level.

#### 3.4.3. Precision and Recovery

The intra-day precision and recovery were calculated for 3 levels: 25 ng/mL (low concentration), 150 ng/mL (medium concentration), and 300 ng/mL (high concentration). Overall, three spiked blood samples were analyzed at each level, and the analysis of each sample was repeated 3 times. The inter-day precision and recovery were calculated from results obtained on 3 consecutive days. Recovery was determined by comparing the obtained concentrations for the spiked blood sample with the nominal value. The recovery was presented as a percentage of nominal concentration.

### 3.5. Preparation of Beverage Samples

**Figure 4:**
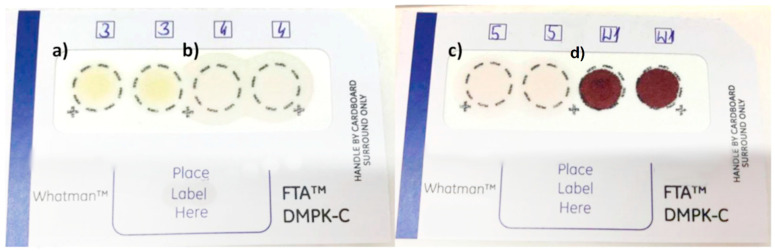
DBS cards with applied beverage samples (**a**) orange juice, (**b**) Coca-Cola, (**c**) rose wine, and (**d**) blood.

In this research, the DBS cards and proposed methodology for identifying the constituents of the DRP (ketamine and flunitrazepam) were used for analysis of three types of beverages: orange juice, Coca-Cola, and rose wine. Spiked samples were containing analyzed drugs and internal standard at the concentration 150 ng/mL. Each stage, such as punching two DBS discs, calculating blood drop diameter as well as MAE extraction, were performed according to the procedure described in Section 3.4. The obtained results for the analyses are presented in Section 2.2. Figure 4 shows analyzed DBS cards with applied drops of beverage samples.

## 4. Conclusions

Ketamine has a double face because it can be used for medical or criminal purposes. It is important to develop a method that may be useful for identifying it in both cases. Therefore, we successfully developed a novel DBS/MAE/CE-TOF-MS method especially for identification of ketamine in different materials. The outcomes from the presented study show that DBS with CE-TOF-MS could constitute a good method for the identification of ketamine in biological material—blood—and in various types of beverages such as orange juice and rose wine. Moreover, the developed methodology offers micro-invasive sampling by using the DBS method, is in accordance with principles of green chemistry, and allows accurate qualitative and quantitative analysis by using capillary electrophoresis coupled with a mass spectrometer. In addition, DBS/MAE/CE-TOF-MS methodology could be useful as a reference methodology for a novel, fast, colour test. Furthermore, the proposed research could play a critical role in the future in the preparation of an innovative device based on portable micro-capillary electrophoresis enabling quick analysis in clinics or in forensic investigations.

## Figures and Tables

**Table 1:** Validation parameters for ketamine in blood samples.

Parameter	Value
Linearity (ng/mL)	LOQ-300
Slope	0.024
Intercept	1.901
R-squared	0.997
LOD (ng/mL):	
according to Equation (1)	6.5
based on signal-to-noise ratio	6.0
LOQ (ng/mL):	
according to Equation (1)	19.5
based on signal-to-noise ratio	19.8
Intra-day ^1^ precision, CV (%):	
50 ng/mL	11.1
150 ng/mL	8.1
300 ng/mL	6.1
Inter-day ^1^ precision, CV (%):	
50 ng/mL	13.1
150 ng/mL	8.6
300 ng/mL	7.9
Intra-day ^1^ recovery (%):	
50 ng/mL	92.7
150 ng/mL	97.4
300 ng/mL	98.2
Inter-day ^1^ recovery (%):	
50 ng/mL	111.3
150 ng/mL	94.6
300 ng/mL	102.4

^1^*n* = 9 for intra-day; *n* = 27 for inter-day.

## Data Availability

Data sharing not applicable.

## Sample Availability

Samples of the compounds are available from the authors.
